# What Affects Authors’ and Editors’ Use of Reporting Guidelines? Findings from an Online Survey and Qualitative Interviews

**DOI:** 10.1371/journal.pone.0121585

**Published:** 2015-04-15

**Authors:** Thomas Fuller, Mark Pearson, Jaime Peters, Rob Anderson

**Affiliations:** 1 Collaboration for Leadership in Applied Health Research and Care (CLAHRC) for the South West Peninsula, University of Exeter Medical School, Exeter, Devon, United Kingdom; 2 Evidence Synthesis & Modelling for Health Improvement (ESMI), University of Exeter Medical School, Exeter, Devon, United Kingdom; University Hospital Lausanne, SWITZERLAND

## Abstract

**Objectives:**

To identify and understand, through data from multiple sources, some of the factors that affect authors’ and editors’ decisions to use reporting guidelines in the publication of health research.

**Design:**

Mixed methods study comprising an online survey and semi-structured interviews with a sample of authors (online survey: n = 56; response rate = 32%; semi-structured interviews: n = 5) and journal editors (online survey: n = 43; response rate = 27%; semi-structured interviews: n = 6) involved in publishing health and medical research. Participants were recruited from an earlier study examining the effectiveness of the TREND reporting guideline.

**Results:**

Four types of factors interacted to affect authors’ and editors’ likelihood of reporting guideline use; individual (e.g. having multiple reasons for use of reporting guidelines); the professional culture in which people work; environmental (e.g. policies of journals); and, practical (e.g. having time to use reporting guidelines). Having multiple reasons for using reporting guidelines was a particularly salient factor in facilitating reporting guidelines use for both groups of participants.

**Conclusions:**

Improving the completeness and consistency of reporting of research studies is critical to the integrity and synthesis of health research. The use of reporting guidelines offers one potentially efficient and effective means for achieving this, but decisions to use (or not use) reporting guidelines take many factors into account. These findings could be used to inform future studies that might, for example, test the factors that we have identified within a wider theoretical framework for understanding changes in professional practices. The use of reporting guidelines by senior professionals appears to shape the expectations of what constitutes best practice and can be assimilated into the culture of a field or discipline. Without evidence of effectiveness of reporting guidelines, and sustained, multifaceted efforts to improve reporting, little progress seems likely to be made.

## Introduction

Variable and incomplete reporting of health and medical research is recognised as a significant and common problem that contributes to wasted research and other resources.[1–3] Poorly reported studies cannot be replicated, nor can results be compared with existing knowledge or easily included in evidence synthesis. Reporting guidelines (checklists, flow diagrams, or explicit text guiding authors in reporting research) have been developed and introduced as an innovation to partially redress this.[4]

While there is no overarching, systematic strategy to disseminate reporting guidelines, the EQUATOR Network aims to raise awareness about the need for more transparent and complete reporting—particularly through the wider use of reporting guidelines. It has amongst other features and functions, an up to date repository of reporting guidelines. Given that over 200 reporting guidelines have been published [5] the EQUATOR Network is a valuable resource for users and potential users of reporting guidelines.

Research suggests that there are variable levels of uptake of reporting guidelines [6]; that reporting guidelines are sometimes misused by authors [7]; that the fidelity of endorsement of guidelines is questionable [8,9]; and, that few have been evaluated.[8,10] Where they have been evaluated, reporting guidelines such as the Consolidated Standards of Reporting of Trials (CONSORT) [9,11,12] have been shown to be effective in improving the reporting of randomised controlled trials (RCTs). There is also some evidence to suggest that the Transparent Reporting of Evaluations with Nonrandomized Designs (TREND) Statement [13] might improve the reporting of studies with non-randomised designs.[14]

Little is currently known though why some reporting guidelines are used more frequently than others, and what factors affect authors’ and editors’ decisions to use reporting guidelines. Authors and editors have been surveyed about many issues (e.g. ethics [15,16], training [17,18], publishing [19–21], outcome reporting bias[22]) and, on one occasion, use of reporting guidelines [23]. In that instance, six editors of journals that endorsed CONSORT and one editor of a journal that did not endorse it were interviewed. Shamseer et al concluded that a more “active” approach should be taken to improve the implementation of reporting guidelines by authors, editors and peer reviewers by targeting knowledge, beliefs, and motivations as well as developing resources to enhance and/or facilitate reporting guideline use.[23] A recent cross-sectional study reported that authors suggested that journal endorsement and the incorporation of reporting guideline checklists into manuscript submission software would facilitate use of reporting guidelines.[24] The time it took to complete checklists, as well as any additional complexity that it might add to a researcher’s workload were predicted to be barriers to use.[24] Others have suggested that use of reporting guidelines is hampered by: word limits; lack of awareness; habit; that journals do not *require* or enforce their use; and, a belief that using reporting guidelines is overly burdensome.[25–27]

In summary, there is limited evidence of reporting guidelines’ effectiveness as conducting these studies is challenging. Additionally, little is known about factors that promote or inhibit authors’ and editors’ use of reporting guidelines. These issues limit the effectiveness of reporting guidelines in helping to address the problem of incomplete reporting of research. The overall objective of this study was to examine factors that affected authors’ and journal editors’ use of TREND and/or other reporting guidelines in the event that they had not used TREND. Improved understanding of what factors affect the use of reporting guidelines could underpin and improve dissemination and implementation strategies for the greater use of reporting guidelines.

## Methods

An overview of the methods is presented here, as further details can be found in the study protocol [28] and in S1 File. This was a follow-up study to one specifically examining the use and impact of the TREND Statement. However as the initial study included authors of articles and editors of journals that had not necessarily used TREND but could also have used additional or other reporting guidelines we asked participants about use of these guidelines as well as TREND. Our aim was to begin to identify factors and conceptualise how they might relate to each other rather than identify a set of factors that definitively explain reporting guideline use.

This study was conducted in two phases. In the first phase we collected quantitative data from authors and editors via an online survey about perceived barriers and facilitators of reporting guideline use. In the second phase, we conducted semi-structured qualitative interviews to obtain detailed information about the context and decision-making processes that affected the use of reporting guidelines.

### Ethics Statement

Ethics approval to conduct the study (including the procedures to obtain informed consent) was obtained from the Research Ethics Committee of the University of Exeter Medical School, United Kingdom (approval number: Jun12/CA/159). Ethics approval was given for participants to indicate their consent via an online form prior to accessing the online survey. Participants who did not indicate their consent were unable to access the survey. S2 File shows the information and consent forms for the online survey.

Potential participants for the interviews (as identified from responses to the online survey) were contacted by email in June 2013 to invite them to participate in a semi-structured interview. The email included, as attachments, an information sheet (including interview topic guide) and a consent form. Participants then returned, via fax or email, signed consent forms prior to the interview.

This research is reported in accordance with the Checklist for Reporting of Results of Internet E-Surveys (CHERRIES; see S3 File)[29] and Consolidated criteria for Reporting of Qualitative research (COREQ; see S4 File) guideline.[30]

### Online author and editor survey

#### Sample and recruitment

The sample of authors and editors was drawn from the 47 primary research studies and 47 comparator papers used in an earlier project examining the uptake and impact of TREND.[14] All corresponding authors, and when possible, a second co-author from papers included were invited by email to complete an online survey.

Editors-in-chief and a second editorial staff member of those journals included in the earlier study were also invited to participate.[14] The editors’ journals were mainly within medicine, psychology, public health and nursing. Two email reminders were sent to all prospective participants.

#### Data collection

The online survey platform was hosted by Bristol Online Survey (www.survey.bris.ac.uk), and open between 16 January and 28 May 2013.

#### Development and content of the surveys

Survey content (see S2 File), was developed from the literature on reporting guidelines [7,24,31], diffusion of innovations [32], and the findings of our earlier study.[14] In recognition of the fact that potential participants might have had some knowledge and experience of using TREND and/or other reporting guidelines, and as a limitation of the functionality of the survey program, three versions of the survey were developed. Specifically, versions were developed for people: without knowledge of reporting guidelines; those who had used reporting guidelines other than TREND; and, for people who had used TREND. Participants were asked questions about their experience of using TREND and other reporting guidelines and perceived barriers and facilitators of the use of reporting guidelines. The wording of the questions was designed to be relevant to either authors or editors. Participants were also invited to take part in a semi-structured interview.

Participants used five point Likert-type scales to indicate their responses to questions (e.g. a scale of 1–5, where 1 is "not very useful", 3 is a “neutral” response, and 5 is "very useful"). A “don’t know” response option was available. Open questions designed to capture qualitative data about reasons for use of reporting guidelines were also included.

#### Data analysis

As the online survey required all questions to be answered there was no missing data. Descriptive statistics were calculated using SPSS v 21.

### Semi-structured interviews

#### Sample and recruitment

Eleven people (five authors and six editors) from the online survey accepted the invitation and were available to participate in a semi-structured telephone interview.

#### Data collection

TF conducted, recorded and transcribed all interviews. Participants were given the opportunity to review the transcript of their interview.

#### Interview schedule

We developed, refined, and piloted the interview schedule with health services researchers with a recent publication record in peer-reviewed journals. The final set of core interview questions asked authors and editors:

- How effective participants thought reporting guidelines were as an intervention to improve the standard of reporting in journals,
- What participants’ peers think about reporting guidelines,
- Why they think reporting guidelines are not more widely used,
- What they think could be done to increase journals’ use of reporting guidelines,
- What could be done to change reporting behaviour of authors and editors respectively,
- How words like “should” and “encourage” in instructions to authors are interpreted,
- How the decision to use reporting guidelines was reached and what else was happening at the time of deciding to use reporting guidelines.

The precise wording of some questions, when feasible, was also tailored to increase relevance and specificity for respective participants. For example, journal editors were asked: “What do your peers at [journal name] and other journals in your field think about reporting guidelines?” Additionally, dialogue was encouraged to allow for additional, unscripted questions and opinions to arise and be expressed during interviews. At the end of the interview, participants had the chance to ask questions and make additional comments.

#### Data management and analysis

We conducted a thematic analysis of the interviews.[33] It involved discussions among the research team and an iterative process through which interpretations of the data and evolving themes were questioned and monitored. This occurred over six phases:

1. Familiarisation through transcribing the interviews and reading of the transcripts.
2. Developing provisional codes and labelling data associated with them.
3. Collating the initial codes into a provisional set of themes.
4. Reviewing and checking potential themes within the data against coded extracts and the complete data set.
5. Refining of themes to ensure that they accurately discriminated between each other and conveyed the key messages from the interviews. Data contradicting the themes were also specifically searched for to establish their robustness.
6. Final refinement to names, definitions and the scope of themes.

This process was documented using Microsoft Excel, Mind Genius and Nvivo 9 software.

## Results

The results of this study are presented in two sections; first the online survey results and following, the results from the semi-structured interviews. Within the section of results from the online survey we also present responses specifically related to TREND.

### Online survey

#### Response rate and sample characteristics

Authors: The overall response rate (i.e. those who started and completed the survey) for authors in the study was 32% (56/173), with a slightly higher response rate for those who had used TREND compared to those who had not (35% vs. 30%). That is, 173 authors were invited to participate, 62 commenced the online survey and 56 of these (90% completeness rate) actually completed it. Further details of authors invited to participate are provided in Table A in S5 File. Authors mainly were from North America, had considerable research experience (median = 15 years; range = 3–45 years), held senior academic positions and worked in public health or psychology. Most authors were *not* aware of the EQUATOR Network (51/56, 91%).

Editors: Of the 159 editors invited to respond to the online survey, 52 commenced it and 43 actually completed it yielding an overall response rate of 27% (43/159) and survey completeness rate of 83% (43/52). See also Table B in S5 File. Editors who responded were mostly from the United States of America (16/43, 37%) and United Kingdom (15/43, 35%), in senior positions (38/43, 88%), and most frequently worked in medicine (12/43, 28%).

Nearly three quarters of editors (72%) indicated that they concurrently held academic or clinical roles while working less than half the time as editors. Responding editors had considerable research experience (median = 21 years; range = 0–42 years) and editorial experience (median = 9 years; range = 0–30 years). Nearly all were members of professional membership organisations (40/43, 93%) and less than half (19/43; 44%) had heard of EQUATOR.

**Fig 1:**
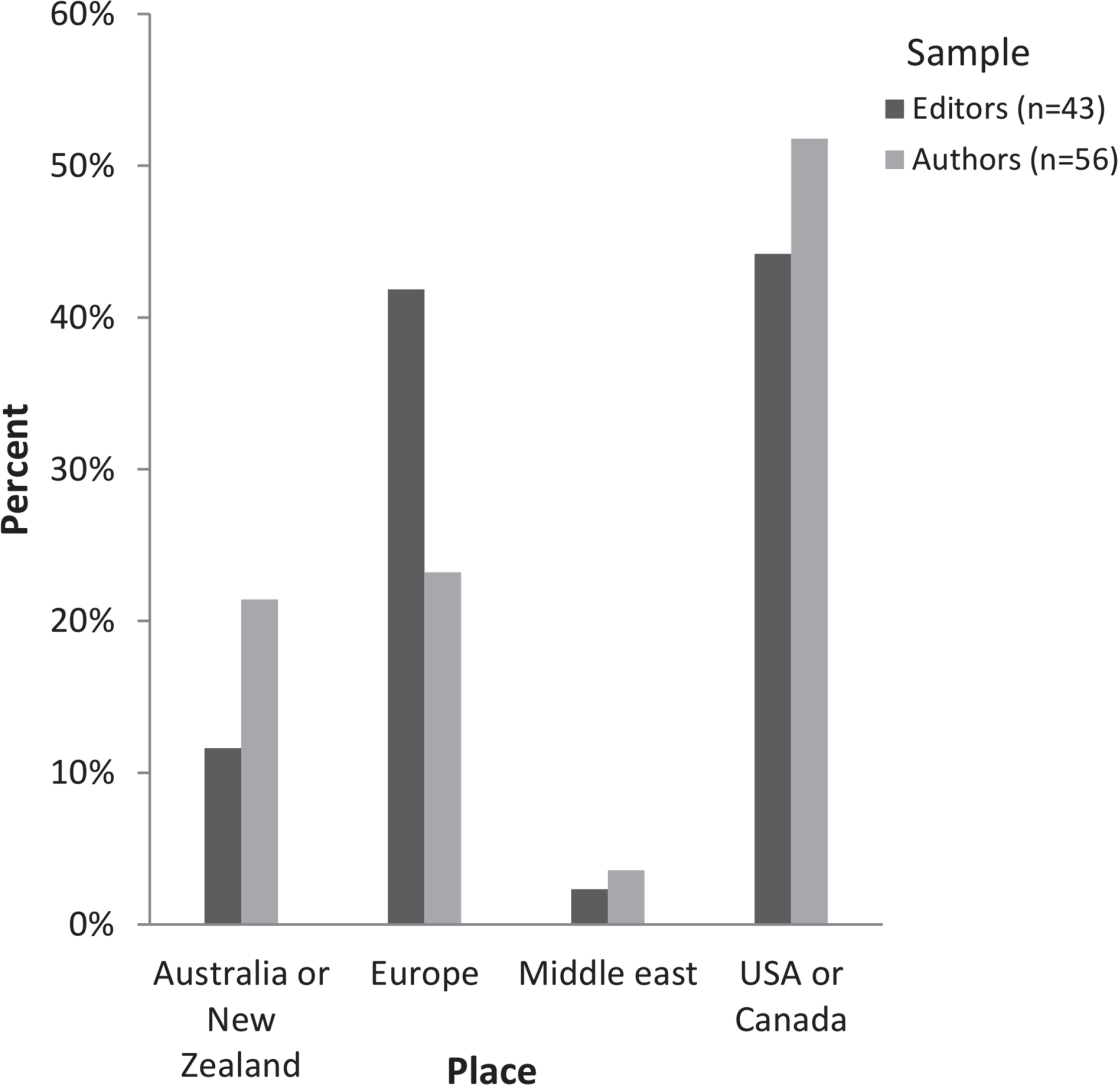
Authors’ and editors’ place of employment.

**Fig 2:**
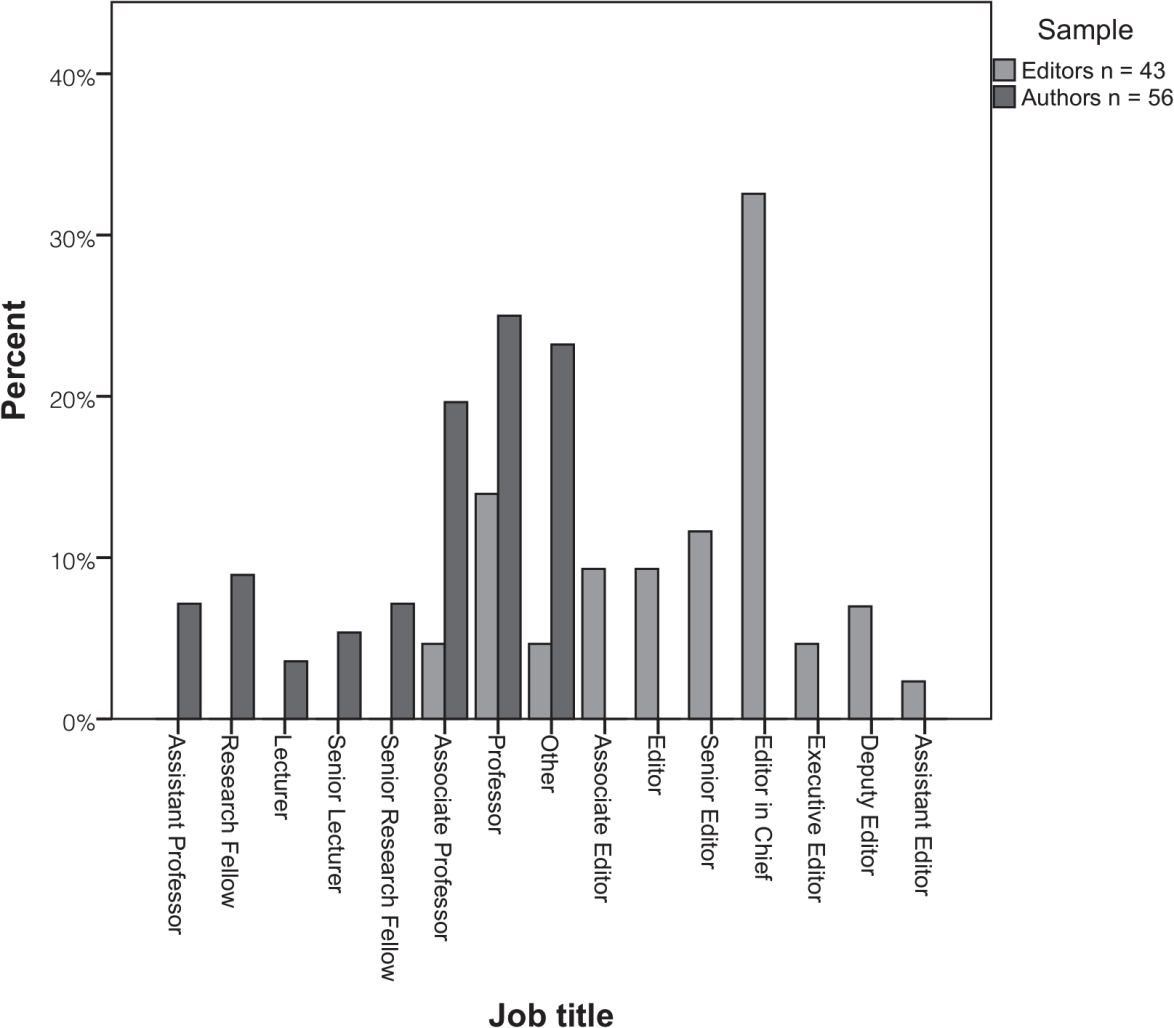
Authors’ and editors’ job titles.

**Fig 3:**
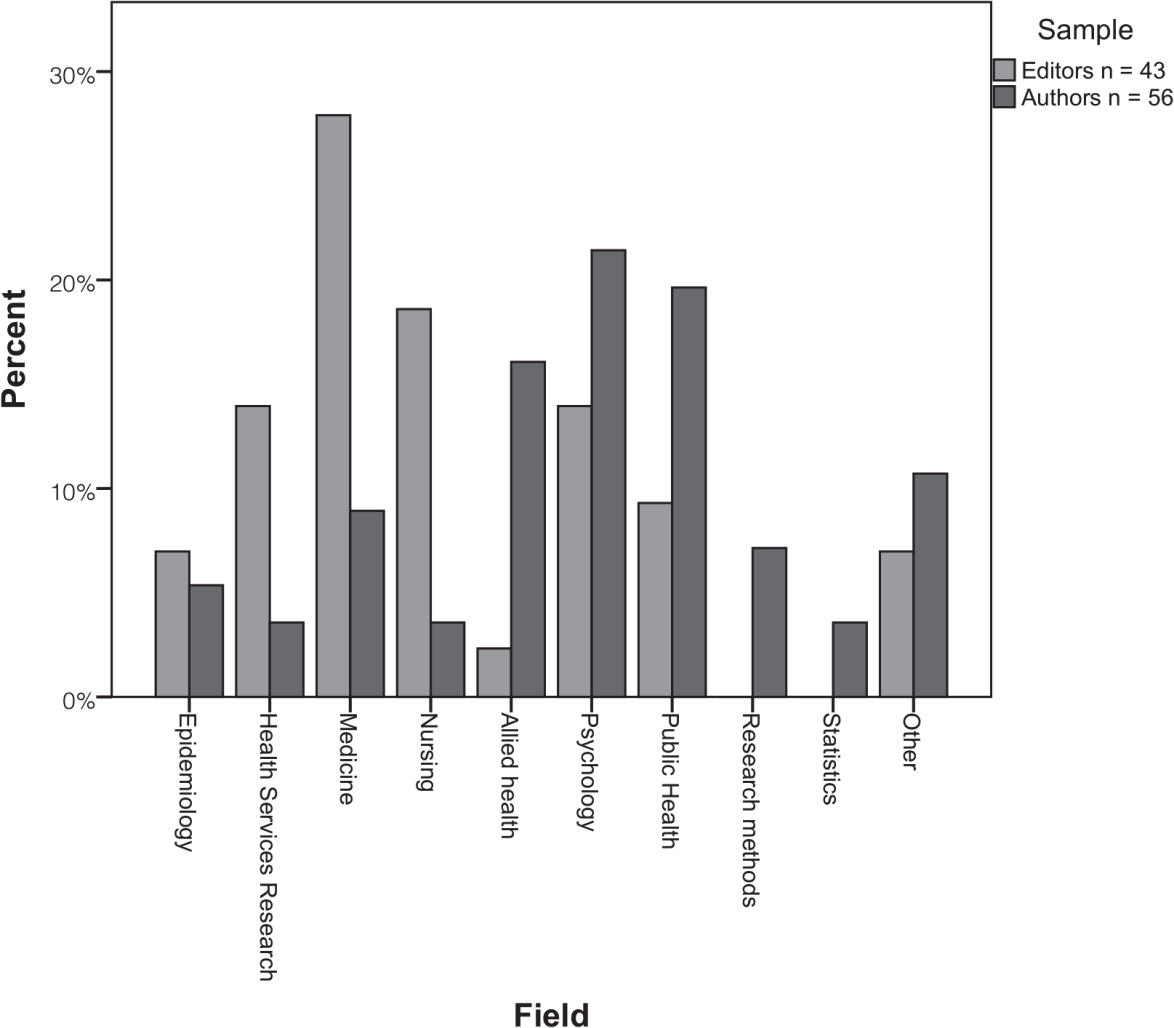
Authors’ and editors’ field of expertise.

Author and editor characteristics are displayed in Figs 1–3.

#### The perceived need for, and familiarity with reporting guidelines

**Fig 4:**
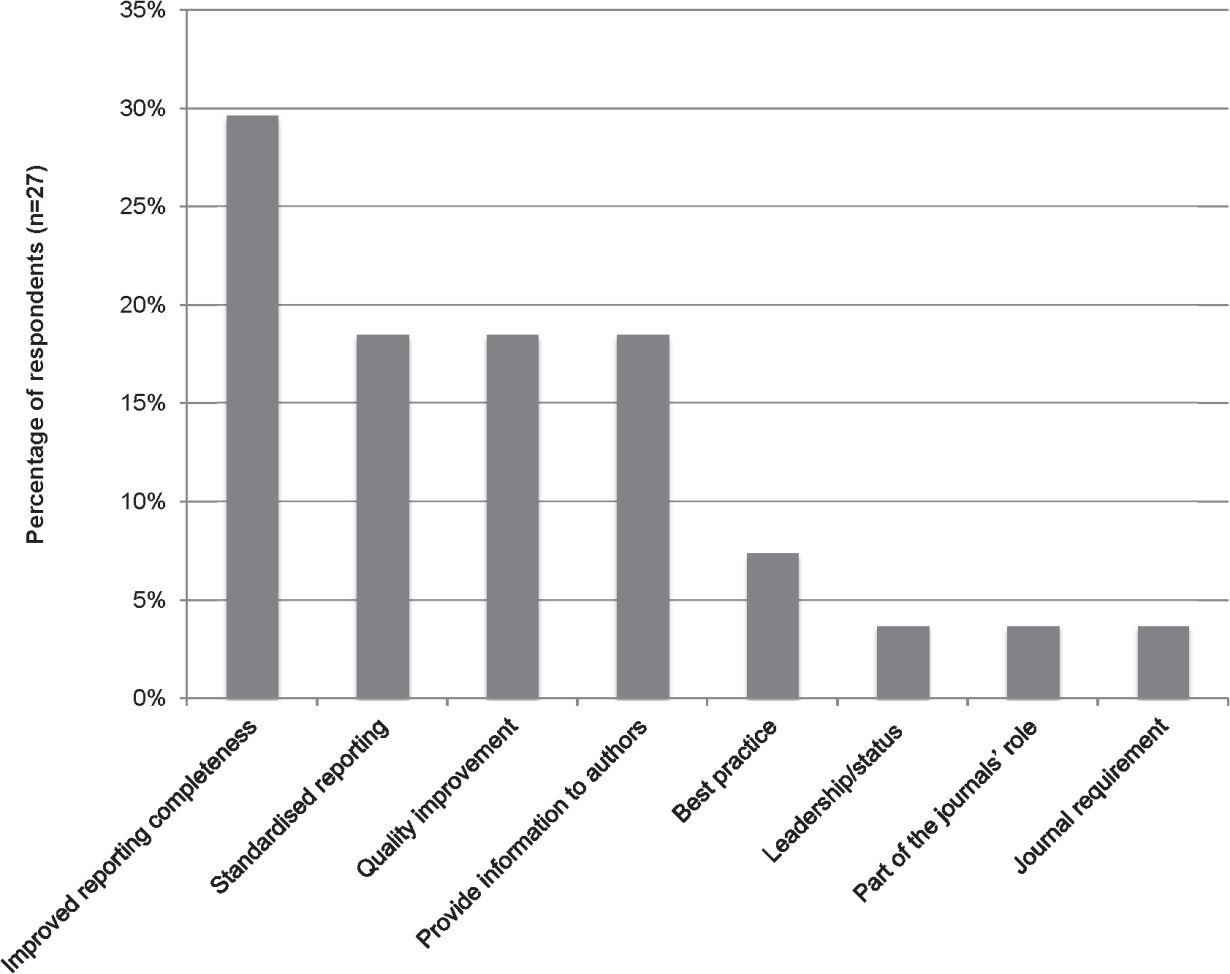
Authors’ and editors’ awareness of selected reporting guidelines.

Both authors and editors believed that information omitted from journal articles was common (authors: 46/56, 82%; editors 31/43, 72%) and represented a problem for consumers of research (authors: 50/56, 89%; editors 37/43, 86%). Most authors and editors were familiar with the CONSORT reporting guideline, and more editors than authors were familiar with a greater range of reporting guidelines (Fig 4).

Most editors believed that all authors (and at all stages of research, especially/including design and reporting), journal editors (especially when considering to send a paper for review) and peer reviewers should use reporting guidelines They also indicated that the responsibility of overseeing the use of reporting guidelines should not be attributed to ethics committees or funding bodies. (See Tables C, D and E in S5 File).

Over 60% (27/43) of editors indicated that their journal referred to reporting guidelines, and 12% (5/43) admitted that they did not know whether or not reporting guidelines were referred to in their instructions to authors.

#### Reasons for and factors affecting use of reporting guidelines

**Fig 5:**
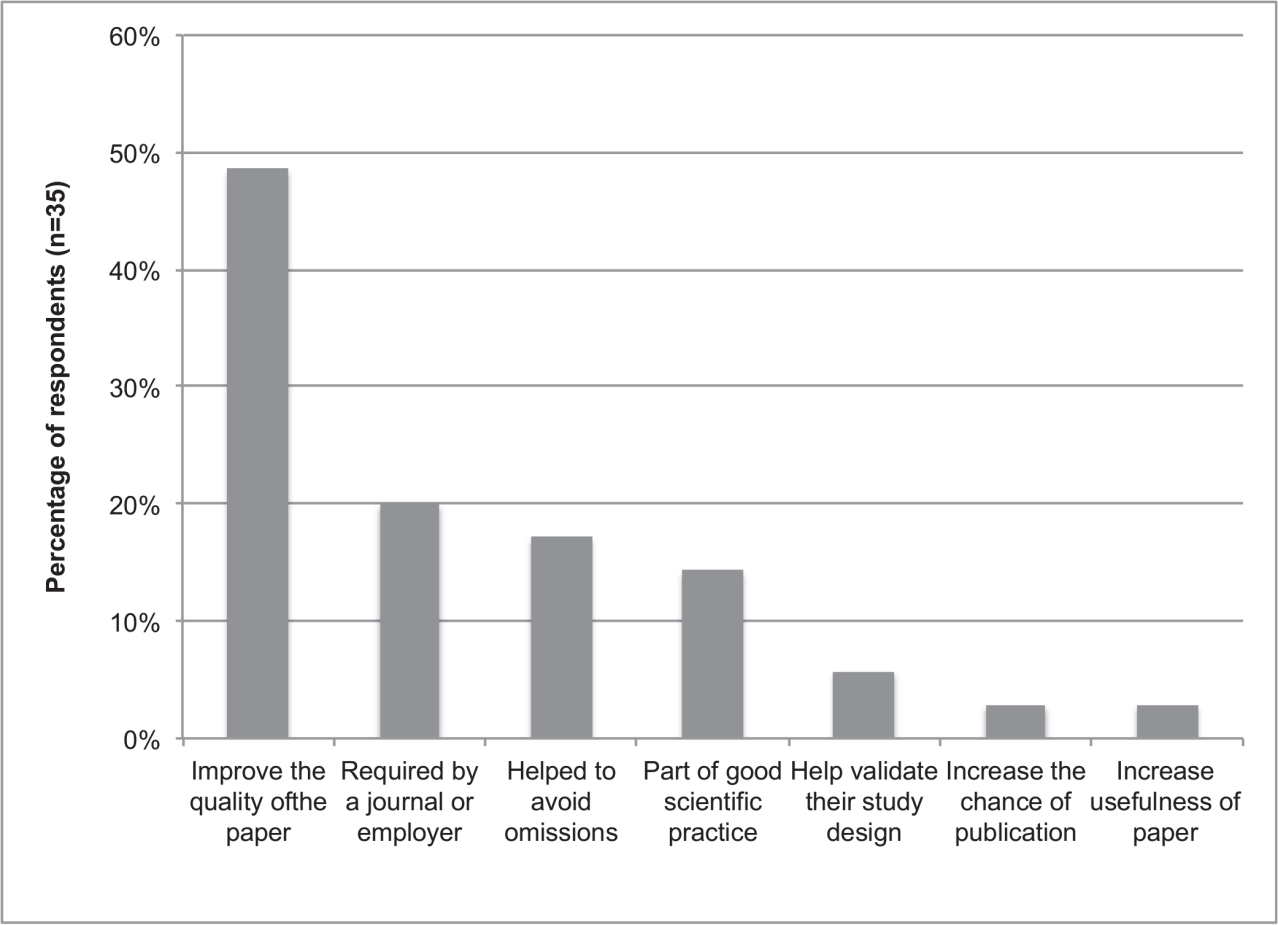
Authors’ reasons for using reporting guidelines.

Authors who had used reporting guidelines (n = 35) reported doing so for a variety of reasons (Fig 5). Participants could make multiple selections from response options and/or indicate their reasons for using a reporting guideline in a free-text field. The most common response was that using reporting guidelines would improve the quality of their manuscript. Authors also indicated that, overall, they believed an environment that was conducive to reporting guideline use was important. For example, journal instructions to authors should be aligned with reporting guidelines and that explanatory reference material should be available to authors to consult when using reporting guidelines if they need guidance. Authors in this sample broadly disagreed with many hypothesised barriers to reporting guideline use (e.g. that reporting guidelines took too much time to complete). (Further details are provided in Tables F and G in S5 File.)

There were no differences between the responses of those authors who had used TREND and those who had not, except for whether transparency of guideline development was important. Authors who had used TREND had disagreed with the statement, while comparator authors thought it was important. There were moderate levels of agreement supporting statements that journal reporting requirements should be aligned with reporting guidelines and that reporting guidelines should be complemented by other strategies to improve reporting completeness. Although most authors reported not being aware of the EQUATOR Network, they indicated that they did not have difficulty locating reporting guidelines. Authors did not consider the evidence base for a reporting guideline when deciding to use one or not. Authors also disagreed with the statement that reporting guidelines take too long to use and they displayed ambivalence about other suspected barriers to reporting guideline use. That is, that they are too prescriptive, that journal instructions are ambiguous or confusing and that journal requirements are considered more important than following reporting guidelines.

Authors indicated that the availability of supporting resources or reference material such as websites or explanation and elaboration documents was important when considering using reporting guidelines, as was use and endorsement of reporting guidelines from professional societies. Authors did however indicate ambivalence about attending courses, workshops or lectures on reporting guidelines affecting their decisions to use reporting guidelines.

**Fig 6:**
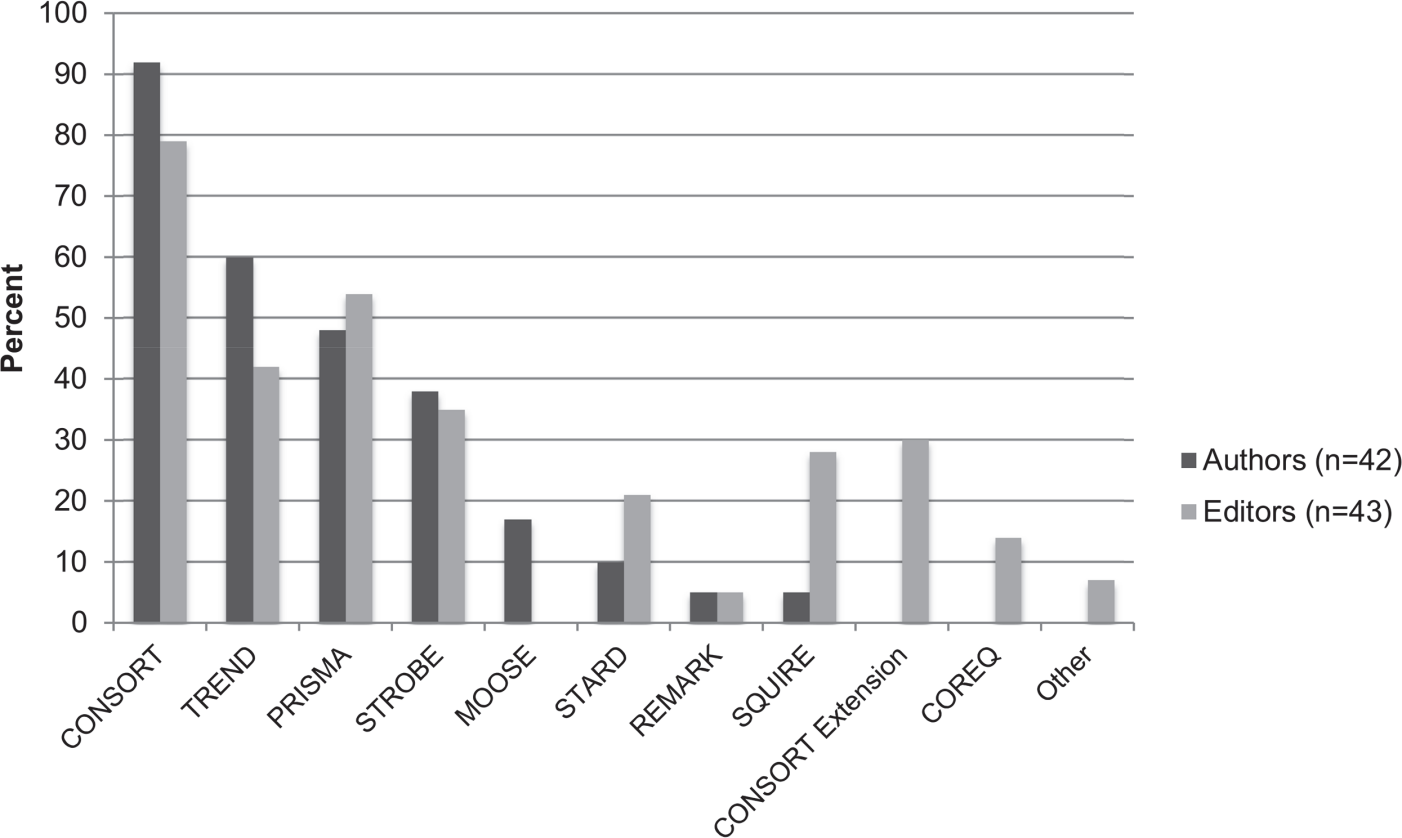
Editors’ reasons for using reporting guidelines.

Fig 6 shows the reasons editors gave for using reporting guidelines. Most editors indicated that they used reporting guidelines in their journals to improve the reporting completeness of manuscripts they received.

Editors, like authors, agreed that endorsement of reporting guideline use by journals and professional societies, and use by colleagues was important. Editors also moderately disagreed with commonly hypothesised reasons for not using reporting guidelines such as taking too much time or that they are too prescriptive. Unlike authors, editors considered the evidence base for reporting guidelines when considering using them or not. (Table 1)

**Table 1:** Editors’ Level of Agreement with Statements Relating to Use of Reporting Guidelines.

	**N**	**Median**	**Min**	**Max**
Importance of journal to promote use of reporting guidelines	40	5	1	5
Importance for improving reporting completeness that authors follow reporting guidelines	40	5	1	5
Importance of reporting guidelines for peer reviewers when reviewing articles	40	4	1	5
Importance that there is a mechanism for giving feedback to reporting guideline developers	41	4	1	5
Editorial board should consider evidence for reporting guideline before including them in instructions to authors	38	4.5	1	5
Transparency of development of reporting guidelines is important when considering use	40	4	2	5
Enforcing the use of reporting guidelines is too time consuming	40	3	1	5
A reporting guideline is too prescriptive for authors when writing up a study	39	2	1	5
Meeting the requirements of the journal is more important than using a reporting guideline	41	3	1	5
There aren't relevant reporting guidelines for my journal’s field of research	37	2	1	5
Importance of website with explanatory information	40	5	2	5
Importance of an explanation and elaboration document	40	4	3	5
Importance of courses, workshops or lectures to support the use of reporting guideline	39	3	1	5
Importance of endorsement of reporting guideline from professional societies	40	4	1	5
Importance of use of reporting guideline by peers	40	4	1	5

Scale of 1 (no agreement) – 5 (strong agreement), with 3 being “neutral”.

Editors indicated a moderate level of agreement with statements that reporting guidelines need to be endorsed or promoted by journals themselves and professional societies—suggesting that merely publishing reporting guidelines is inadequate for the widespread uptake and use of reporting guidelines. The use of reporting guidelines by one’s peers was also seen as a factor that would influence the use of reporting guidelines. Similarly, there was agreement that resources supporting the use of guidelines, such as explanation and elaboration documents and websites were important for users of reporting guidelines.

The fact that editors were ambivalent about the importance of lectures and/or workshops to support the use of reporting guidelines but valued explanation and elaboration documents and guideline websites is consistent with the view that those resources are adequate to facilitate the effective application of reporting guidelines.

Of the 11 editors whose journal did not use or promote reporting guidelines, seven indicated that evidence of the effectiveness of reporting guidelines would make them more likely to consider adopting them in the future. Other factors that would influence their use of reporting guidelines included: evidence showing improved journal status or metrics associated with use of reporting guidelines (5/11); a requirement of publishers (3/11); and, endorsement of the use of reporting guidelines from editorial colleagues.

### TREND specific information

#### Authors

Authors who were aware of TREND indicated that they had learned of it through a range of ways including: through colleagues and mentors; websites; journal articles that had either used or were about TREND; in the course of writing a paper or conducting a study that TREND was relevant for; and, discovering it in the course of conducting a literature review.

Authors reported the strengths of TREND were related to the: guidance that it provides for what to write (13/28, 46%); consistency/standardisation of content (7/28, 25%); relevance to evaluations of behavioural and public health interventions (3/28, 11%); and, validation of non-randomised designs (2/28, 7%). Three “don’t know” responses were also given.

Of these perceived strengths, only one relates specifically to TREND—that having a reporting guideline specifically for studies with non-randomised designs in some way validates the study design.

When authors were asked what they perceived the main limitations of TREND to be, most, (61%, 17/28) responded by writing, “don’t know” (or equivalent). Limitations that were mentioned included perceptions that it was too: medically orientated (2/28); inflexible/rigid (2/28); general (2/28); that it had become out dated (1/28); was generally unknown (1/28); had insufficient detail (1/28); needed further development (1/28); and, that use of it was only suggested rather than required (1/28). It is not clear whether these perceived limitations also acted as barriers to TREND’s use. However, the fact that the response rate to this question was so low, suggests that people who have used TREND, are not familiar enough with it to make comments on its weaknesses.

#### Editors

Editors who had heard of TREND (n = 18) reported in free-text fields that they had learned of it through colleagues, reading articles, publishers, the EQUATOR Network, in the course of teaching and their journal’s requirements. Table 2 summarises what editors’ perceived to be strengths and limitations of TREND.

**Table 2:** Editors’ perceptions of TREND.

**Perceptions of TREND**		**N = 18**	**Percentage of responses**
Strengths	Relevance to non-randomised designs	6	33
	Standardisation of/consistency in reporting	5	28
	Completeness of reporting	3	17
	Don’t know	3	17
	Improves clarity/transparency	2	11
	Provides structure	1	6
	Helps to spot omissions	1	6
Limitations	Don’t know	4	18
	Lacks clarity/specificity	3	17
	Not widely known or universally accepted	3	17
	Excessive requirements	2	11
	Not revised	1	6
	Irrelevant to journal	1	6
	Stigma against non-randomised designs	1	6
	None	1	6

Note: Responses are only from editors who had actually used TREND.

In summary, participants believed that incomplete reporting to be a common and important problem; thought that use of reporting guidelines would lead to a better quality paper, but that there was a wider range of barriers to their use than previously documented.

### Semi-structured interviews

#### Sample characteristics

The authors (two female, three male) and editors (four females, two males) who participated in the interviews were very experienced and held relatively senior positions. Specifically, authors had considerable amounts of research experience (median = 13 years; range = 3–45 years), held positions as Professor, Senior Director and Research Fellow and were from the United States of America or Australia. Editors had considerable editorial experience (median = 20 years; range = 10–24 years) and research experience (median = 18.5 years range = 0–30 years), respectively. Five editors were from United Kingdom and one from the United States of America and held positions as Senior Editors, Editor-in-Chief, Professor, Executive Editor and Editor (note, in some instances, people held multiple positions).

In response to the question about perceived effectiveness of reporting guidelines, all interviewees were positive in their responses. Though reporting guidelines were seen to be effective, there was also acknowledgment of the difficulties associated with use, implementation and development.

#### Emerging themes

Themes and concepts discussed by participants were grouped according to whether they related to:

1. Individual factors—a person’s qualities and beliefs that affect their likelihood to use reporting guidelines (e.g. motivations and beliefs);
2. Professional culture and expectations about how work should be conducted (e.g. expectations that RCTs should be reported using CONSORT);
3. Environmental factors—broader external conditions that affect the research environment or influence practices and likelihood to use reporting guidelines (e.g. journal or regulatory agencies’ policies); and
4. Practical factors—perceived or actual constraints that directly impact on the likelihood of using reporting guidelines (e.g. inability to find reporting guidelines).

Table 3 provides example of issues that potential users of reporting guidelines considered.

**Table 3:** Issues faced by authors and editors when considering using reporting guidelines.

**Factor**	**Examples of issues considered**	
	**Authors**	**Editors**
**Individual**	Perceived pros and cons of using reporting guidelines; beliefs about use of reporting guidelines, knowledge and prior experience of using reporting guidelines.	Perceived pros and cons of using reporting guidelines; knowledge of and prior experience of using reporting guidelines.
**Professional culture**	Are principal investigators, leaders in the field, or peers using reporting guidelines when publishing research on a similar topic/study design?	What are other journals in the field doing? What are the publisher’s expectations?
**Environmental factors**	Does the journal only suggest or actually require submission of a reporting guideline checklist?	What are the requirements of the publisher, funding or (relevant) regulatory agency?
	If required, is it at the point of manuscript submission or is it once the manuscript has been accepted?	
**Practical factors**	Is sufficient time available to comply with reporting guideline?	Do the editor, administrative staff or reviewers have the time to check reporting guideline checklists?
	When, in the process of writing or conducting the study, was it realised that reporting guidelines could or had to be used?	Is there support from the publisher to adopt policy of reporting guidelines use?
		What is the evidence base for the impact of reporting guidelines?
		Does the online manuscript submission system support reporting guideline checklist use?

#### Individual factors

It is important to have more than one reason for using reporting guidelines: Analysis revealed that editors and authors consistently indicated that having multiple reasons facilitated reporting guideline use. For example:

Author 3: I think if it was a high impact factor journal and I thought that I would only get one crack at it… I would be more likely to use the CONSORT statement.

and;

Editor 2: We did it [included reporting guidelines in instructions to authors] partly because… it was actually an ambition for the journal to become a much better, a much more significant journal in the field.

Anticipated effort affects use of reporting guidelines: At an individual level, the implementation of a new intervention necessarily requires the modification of an existing practice or the learning of new behaviours. Hence learning or adaptation takes effort above that required to perform a previously routine practice such as writing a paper without using a reporting guideline. As Editor 2 says: “… it is that initial experience [of writing a paper and using a reporting guideline]. I think that initial experience is quite difficult.” The implication is that with experience, using the reporting guidelines and writing up studies becomes easier. This contrasts though with the perspective of the editorial board of another editor, which was concerned that reporting a study well or using a reporting guideline was easy to do, and that requiring authors to use reporting guidelines was patronising—or in their words, “…. this is teaching your grandmother to suck eggs.” The authors, however, spoke of reporting guidelines being difficult to use initially but that the requirement to do so was not patronising.

Bias could affect reporting guideline use: Bias seemed to play a role when editors discussed which reporting guidelines they were likely to use within their respective journals. Given that not all reporting guidelines could be individually referred to by a journal’s instructions to authors (nor would all likely be relevant for any one journal) some editors indicated that they just had to choose or refer to one or two to mention rather than all the relevant ones for their particular journal. For example, one editor described how endorsing and requiring the use of CONSORT and PRISMA was a “no brainer”. For other guidelines though—especially relating to qualitative research—the journal had adopted a more “cautious” approach. PRISMA does not share the same level of empirical support as CONSORT (it is a more recent guideline after all), but because systematic reviews produce levels of evidence that minimise risk of bias in assessing interventions [34], perhaps editors are more willing to endorse it than reporting guidelines for qualitative research. This positive bias towards PRISMA is in turn likely influenced by broader institutional and environmental factors such as the drive by the Cochrane Collaboration and other groups to improve the quality of systematic reviews.

It is also worth remembering that one editor who had used TREND responded in the survey, that “stigma against non-randomised designs” was a weakness of it. Additionally, the survey data indicated that editors do consider how a guideline was developed when deciding to use reporting guidelines, yet none who were interviewed referred to this.

#### Professional culture

Some authors and editors used reporting guidelines because it was part of routine practice—it was what was implicitly expected of them to do, either in the reporting of their study or administration of the journal. For example:

Editor 3: most good quality systematic reviews that they read in our journal, as well as in other journals seem to follow this general format [of using PRISMA]. I really suspect that it's not related all that much to what we say in our info for authors—that it's more the general milieu of high quality systematic reviews that they're exposed to now.

Another editor commented on the responsibility that universities have, as institutions of education and employment, to directly influence the practices of authors. Complementing this, two authors referred to the influential roles of principal investigators and experts in guiding practices. In contrast to these examples, one editor discussed how the lack of expectation to use reporting guidelines actually inhibits the use of them when reporting qualitative research.

#### Environmental factors

Three factors affecting the publishing environment and use of reporting guidelines emerged from the interviews. Interviewees did not mention environmental factors relating to publishers, funding bodies or other policy drivers.

Poor dissemination strategies inhibit uptake of reporting guidelines: Both authors and editors described how they thought that a poor dissemination strategy by authors of reporting guidelines had inhibited uptake of them. Furthermore, the absence of any on-going promotion of most of the reporting guidelines contributed to authors in particular, forgetting to use them or not being aware of them.

Editor 7:… their almost universal weakness is that they think that just doing them and announcing them [reporting guidelines] will be the same as dissemination of them…

On the other hand there was the perspective that reporting guidelines had been disseminated well, but that it is actually other factors that prevent their use.

Editor 6: … the answer that seems to come back from the CONSORT group when they approach editors, is that editors say, "I've got enough to do…I haven't got the support from the publisher… they basically don't quite get it and don't have the resources or the time…

This quote illustrates how editors might well be aware of a range of guidelines, but due to limitations on their time or a lack of support from their publisher they are unlikely to do more about their implementation.

Enforcement of editorial policy to use reporting guidelines works (especially in high impact journals): Authors and editors spoke of enforcement policies only in relation to authors. This suggests that editors of highly reputed journals are in a position to influence the use of reporting guidelines. Comments from authors also suggested that the status of the journal might act as a mediating variable regarding their adherence to a journal’s requirements.

Absence of evidence inhibits uptake of reporting guidelines: Editors referred to a sense of scepticism amongst colleagues and editorial boards that reporting guidelines were effective in improving transparency in research reports. For example:

Editor 2: I think there’s huge variation in awareness to be honest. I think people who are most aware are most in favour with the possible exception of reporting guidelines around qualitative research where I think there is much less consensus. I think that is an area where there is a lot of awareness of reporting guidelines but less clarity that people tend to think that they are a good thing.

Despite the lack of evidence though, editors in favour of reporting guidelines were often able to reach a compromise with editorial boards to at least “suggest” the use of reporting guidelines rather than mandate their use for all submissions of a given type of research.

#### Practical factors

Having time to use or learn about reporting guidelines is important:

Editors spoke more than authors about the effects of not having enough time to know about or use all the relevant guidelines. This suggests that *if* authors are motivated to use reporting guidelines then this means that they see it as part of the writing process and part of being a researcher. It also highlights the additional responsibilities and demands of editing a journal.

Removal of obstacles to reporting guideline use: Both editors and authors spoke of the need for removing barriers to the use of reporting guidelines. They reasoned that anything that contributed to making it difficult to find or use reporting guidelines would decrease the likelihood of their use. For example, authors spoke of how having clear instructions to authors about the expectation to use a reporting guideline—preferably with hyperlinks to the reporting guideline itself—would make it easier for authors to find and use them. Similarly the relaxation of word limits on manuscripts and/or the ability to provide supplementary files was seen as important by both groups of participants.

Editor 6: …anything that makes life easier for authors, means that it's probably, slightly, more likely to happen.

This need to make it as easy as possible to use reporting guidelines implicitly assumes that most authors are not inherently motivated to apply reporting guidelines in their research. This contrasts with those who are highly motivated to use reporting guidelines and would do so despite obstacles that they encounter.

## Discussion

Understanding why authors and editors might use reporting guidelines is fundamental to increasing the use of reporting guidelines, and ultimately, the completeness of reporting of research. We used quantitative and qualitative methods to obtain complementary data that illuminate some of the determinants of the use of reporting guidelines.

### Principal findings

Editors and authors believed that omitted information is a common and serious problem and, importantly, that reporting guidelines are an effective part of the strategy to improve reporting. Results from the survey and interviews suggest that authors’ and editors’ use of reporting guidelines is largely determined by a combination of individual, professional culture, environmental and practical factors. The influence of some factors (e.g. professional culture) has similar effects for both authors and editors. However, it is likely that practical factors would affect authors and editors differently. Having multiple reasons for using reporting guidelines appeared to be a particularly salient factor in facilitating reporting guidelines use. It implies that authors and editors adopt a rational approach to considering use of reporting guidelines and that, if there are enough advantages for using them, they will do so despite any perceived disadvantages.

Where the use of reporting guidelines becomes an expected part of research practices—as it arguably has for the reporting of RCTs and systematic reviews—self-reinforcement and normalisation occurs. Normalisation of reporting guideline use is likely to occur at different levels and rates for different study designs. That is, at a “micro” level, normalisation of reporting guideline use might occur within an individual or specific research team over a relatively brief period of time. At a “mid” level, normalisation might develop within a particular institution, or, at a “macro” level within a particular field or discipline and over a longer period of time. Wanting or needing to comply with what is considered normal practice can also be seen as having an element of self-regulation and could be formalised through journal or institutional policy.

### Comparison with other studies

Journal endorsement of reporting guidelines as well as the incorporation of reporting guideline checklists into manuscript submission software has been reported [23] and predicted to facilitate and increase use of reporting guidelines.[24] Our results indicate that authors are indeed likely to be influenced to some degree by journal endorsement, though they did not report a particular need for additional practical or instructional materials in the use of reporting guidelines to be provided. Our study, surprisingly, found that authors were relatively unconcerned by the practicalities of actually using a checklist.

Our results were consistent with others that have found that enforcement of editorial policy can play an effective role in changing authors’ behaviours.[35] Incorporating reporting guidelines into early and on-going research methods training has been advocated as a way to improve reporting of studies. [36–38] Evidence from participants in this study supports this claim, suggesting that early exposure to reporting guidelines and prior experience of using reporting guidelines does facilitate their use. The editor survey result indicating that they considered the evidence base of reporting guidelines before endorsing them though is in contrast to anecdotal reports suggesting that editors select reporting guidelines according to perceived relevance to a journal.

### Strengths and limitations

Our study design enabled us to survey and interview a range of experienced authors and editors about their opinions and experience with using TREND and other reporting guidelines. The questions in the semi-structured interviews were also constructed in a way to be relevant to both authors and editors, thus generating data from differing perspectives on a given issue. Furthermore our analysis of the data gives details and examples of the complexities in people’s circumstances when considering using reporting guidelines.

The results of our study should be considered in the context of three limitations that primarily affect the generalisability of the findings. First, we recruited participants from authors who specifically cited the use of TREND or a sample of those who could have used TREND but did not report doing so. Editors were recruited from a sample of journals that publish studies that could have or did use TREND. While we were particularly interested in factors affecting the use of the TREND Statement (this being a complementary study to Fuller et al 2014 [14]) and hence drew from this pool of participants, we are aware that authors and editors respectively do not necessarily limit themselves or their journals to a particular methodology. It is possible that they might have had knowledge of or experience with using other sets of reporting guidelines and this was why we asked about factors that might have affected their use of reporting guidelines other than TREND.

A second limitation was that we obtained a relatively low response rate to the online survey. While our response rates for authors (32%) and editors (27%) was close to the range reported in a meta-analysis of web based surveys (34–39%)[39] and Shamseer et al [23] it was lower than that of other studies recruiting editors and authors. [15,16,40–42] A third limitation relates to the relatively small and homogeneous (mostly experienced and senior authors and editors) sample of people responding to the online survey.

Taken together, these limitations produced a sample of participants whose views on reporting guideline use is unlikely to be representative of all authors and journal editors. The demographic characteristics of the sample though are similar to that reported for surveys of medical [41] and nursing [40] editors. Nevertheless, we believe the importance of having an entirely representative sample is less significant in an exploratory study examining this new domain of reporting guideline use.

### Conclusions and implications

Our findings represent initial but fundamental research into the reasons why reporting guidelines are or are not used by study authors and journal editors. These findings could be used to inform future studies that might, for example, test the factors that we have identified within a wider theoretical framework for understanding changes in professional practices, such as Normalization Process Theory.[43] Further research could also usefully: assess the relationship between reporting guideline use and time taken to review manuscripts; evaluate the impact on reporting completeness of reporting guidelines that have not yet been examined; or, assess the impact of introducing a mandatory reporting guideline use policy on a journal’s submission rates.

#### Working towards improved reporting of research

Improving the evidence base around the impact of the many unevaluated reporting guidelines should be a priority for reporting guideline authors, developers and promoters. Without underpinning arguments for the dissemination and promotion of the use of reporting guidelines, doubts will remain about the value of endorsing and using reporting guidelines.

In particular, editors of influential journals should be explicit in requiring the use of reporting guidelines at the point of manuscript submission, and, in some way, enforce this. Our results suggest that authors are highly motivated to publish in highly reputed journals and will carefully follow instructions to authors if they perceive that that would potentially increase their chance of publication. In these instances editors should have little fear that a strongly worded policy will significantly affect submission rates.

Leading journals, experts, and educators within health and medical fields are all in positions where they can promote the idea that use of evidence based reporting guidelines is part of being a good researcher. This could be done through: research methods classes; the use and citation of reporting guidelines by experts; and, widespread journal requirements that authors use and submit reporting guideline checklists.

Improving the completeness and consistency of reporting of research studies is critical to the high scientific quality, perceived integrity and effective synthesis of health and medical research. The use of reporting guidelines offers one potentially efficient and effective means by which to achieve this but decisions to use (or not use) reporting guidelines are more complex than this motive implies. Thus it is necessary to create an environment and culture that rewards (directly or indirectly) more complete reporting using existing guidelines. This should in turn lead to significant benefits in evidence syntheses, research, policy and health service delivery.

## Supporting Information

S1 File (DOCX)

S2 File (DOCX)

S3 File (DOCX)

S4 File (DOCX)

S5 File (DOCX)
