# Supplementary material for: What Affects Authors’ and Editors’ Use of Reporting Guidelines? Findings from an Online Survey and Qualitative Interviews
Source: PLoS One. 2015 Apr 15;10(4):e0121585. doi: 10.1371/journal.pone.0121585 (PMC4398362; doi:10.1371/journal.pone.0121585)
Supplement: S1 File — (DOCX) [file pone.0121585.s001.docx]

**S1 File**

## Online surveys

## Online author and editor survey

***Development and content of the surveys***

All potential participants were emailed information about the study, invited to contact the research team if they had any questions about participating, or did not want to participate in the study. Where we received notification of invalid email addresses, an online search for a current email address was conducted. In the event that one was not located, a replacement author or editorial staff member from the same article or journal respectively was selected and invited to participate.

***Data collection***

Participation in and access to the survey was not intended to be open, but, theoretically it could have been accessed and completed by anyone. However, we think this is highly unlikely given that there was a low response rate even from those we were actively trying to recruit.

The “welcome” pages provided details about the study, and informed respondents that cookies would not be used and that all questions were mandatory unless otherwise indicated. A hyperlink to the study protocol and the PenCLAHRC website respectively were included. Respondents were also informed that it was not possible for them to review or return to previously completed pages once they had clicked the “continue” button on a particular page. Participants however could pause and save their progress through the survey and complete it at a later time.

No incentives were offered to potential respondents.

***Functionality of the online survey***

Progress through the survey was indicated by a page counter, up to 21 pages/screens , with the number of questions ranging from one to six per page. The relatively high number of pages for the survey(s) was essentially a by-product of having relatively few questions per page so that respondents did not have to scroll down the page to check to see if they had answered all the questions. It was not possible for participants to leave a page of questions without having completed all the mandatory items.

There were some limitations of the capacity of the Bristol Online Survey that affected the design and functionality of the survey. These included: participants not being able to review their responses once they had progressed or completed the survey; items not being able to be randomised; not being able to use cookies to monitor unique visitors to the survey; and, not being able record page views or monitor IP addresses to detect possible duplicate entries from the same user. Adaptive questioning was used whenever possible in the questionnaire in order to reduce the number of questions for participants.

***Data analysis***

No statistical correction was used to adjust for the composition of the sample.

## Semi-structured interviews

***Data storage***

Digital recordings of interviews were downloaded and stored securely on the University of Exeter network, password protected and transcripts de-identified.

***Additional information to meet the criteria of COREQ-32 checklist***

Domain 1: Research team and reflexivity

2. Credentials: TF (male) completed a Doctorate in Psychology and undertaken training in training in thematic and framework analysis. MP (male), JP (female) and RA (male) have PhDs and are experienced in qualitative and quantitative data analysis and evidence synthesis and between them have at least 30 years research experience.

3. Occupations at time of the study: TF, Associate Research Fellow; MP, Senior Research Fellow; JP, Research Fellow; RA, Associate Professor, University of Exeter Medical School.

4. Gender: See above point 3.

5. Experience and Training: See point 3.

6. Relationship established: No prior relationship with participants was established prior to the interview.

7. Participant knowledge of the interviewer: The interviewer introduced himself to participants and explained the background to the study but did not provide details of his training or background. Publicly available information about the research team is available on the University of Exeter website.

8. Interviewer characteristics: The interviewer and research team are interested in and have conducted research into publication bias in public health research. At the time of the interviews, the research team were undertaking an evaluation of the impact of the TREND reporting guideline. At the time of the interviews, the outcomes of that study were unknown.

Domain 2: Study design

11. Method of approach: Participants were approached via email.

Setting

14. Setting of data collection: TF conducted all the interviews from a private office space at the University of Exeter.

15. Presence of non-participants: No one else was present during the interviews.

Data collection

18. Repeat interviews: No repeat interviews were conducted.

20. Field notes: Field notes were made during and immediately after the interviews.

21. Duration: Interview times were from 18 – 60 minutes. Most were approximately 25 minutes.

22. Data saturation: Data saturation is not discussed.

Domain 3: analysis and findings

Data analysis

28. Participant checking: Participants did not provide feedback on the findings from the interviews.

References

1. Fuller T, Pearson M, Peters J, Anderson R (2014) 10 years on... What impact has TREND had? (accepted American Journal of Public Health).

2. Hopewell S, Altman D, Moher D, Schulz K (2008) Endorsement of the CONSORT Statement by high impact factor medical journals: a survey of journal editors and journal 'Instructions to Authors'. Trials 9: 20-26.

3. da Costa BR, Cevallos M, Altman DG, Rutjes AWS, Egger M (2011) Uses and misuses of the STROBE statement: bibliographic study. BMJ Open 1.

4. Burford BJ, Welch V, Waters E, Tugwell P, Moher D, et al. (2013) Testing the PRISMA-Equity 2012 Reporting Guideline: the Perspectives of Systematic Review Authors. PLoS ONE 8: e75122.

5. Greenhalgh T, Robert G, Macfarlane F, Bate P, Kyriakidou O (2004) Diffusion of innovations in service organizations: systematic review and recommendations. Milbank Quarterly 82: 581-629.
