# Supplementary material for: What Affects Authors’ and Editors’ Use of Reporting Guidelines? Findings from an Online Survey and Qualitative Interviews
Source: PLoS One. 2015 Apr 15;10(4):e0121585. doi: 10.1371/journal.pone.0121585 (PMC4398362; doi:10.1371/journal.pone.0121585)
Supplement: S2 File — (DOCX) [file pone.0121585.s002.docx]

### S2 File

### Author and Editor surveys

### Author Survey……………………………………………………………………………………………………………… p. 1

### Editor Survey …………………………………………………………………………………………………………… p. 16

### Author Survey

Page 1 of 21

**Welcome**

Welcome to this survey about reporting quality and guidelines. The purpose of this survey is to collect information about your views about the quality and completeness of reporting of research studies, and whether or not reporting guidelines might be an effective strategy to improve reporting. 

The survey has received ethics approval (reference: 15/06/159) from the Research Ethics Committee of the University of Exeter Medical School, United Kingdom. The study protocol has been published in BMJ Open and is available here: <http://bmjopen.bmj.com/content/2/6/e002073.full.pdf+html>

Funding for project was provided by the National Institute for Health Research (NIHR) Peninsula Collaboration for Leadership in Applied Health Research & Care (PenCLAHRC). For more information about PenCLAHRC go to: <http://clahrc-peninsula.nihr.ac.uk/>

**Completing the survey**

The survey is completed anonymously, can be saved part way through and takes around 10 - 15 minutes to complete. Questions are mandatory unless marked otherwise.

*Note: once you have clicked on the CONTINUE button your answers are submitted and you cannot return to review or amend that page.*

**Data protection**
All data collected in this survey will be held anonymously and securely.

Cookies and personal data stored by your Web browser are not used in this survey.

**If you need assistance with any aspect of completing the survey or encounter errors with the program, please email** [**t.fuller@exeter.ac.uk**](mailto:t.fuller@exeter.ac.uk)

### 1. Consent

| **1.** I have read the project information sheet and give my informed consent to participate in the study Evaluating the impact and use of the Transparent Reporting of Evaluations with Non-randomised Designs (TREND) reporting guidelines. |
| --- |
| **Yes** |

### 2. Professional background

| **2.** What is your current job title? |
| --- |
| **Assoc. Research Fellow** |
| Research Fellow |
| Lecturer |
| Senior Lecturer |
| Senior Research Fellow |
| Assoc. Prof. |
| Professor |

| **3.** Please provide an estimate of years of research experience. |
| --- |
|  |

### 3. Professional background

| **4.** In which country are you employed? |
| --- |
|  |

| **5.** What is your primary field of expertise? |
| --- |
| Epidemiology |
| Evidence synthesis/systematic reviews |
| Health services research |
| Medicine |
| Nursing |
| Physiotherapy |
| **Psychology** |
| Public Health |
| Research Methods |
| Social Work |
| Statistics |

| **6.** Are you a member of any professional organisations, societies or colleges? |
| --- |
| No |
| **Yes** |
| **6.a.** Which professional organisations or societies are you member of? |
|  |

### 4. EQUATOR Network

| **7.** Are you aware of the EQUATOR Network? |
| --- |
| No |
| **Yes** |
| **7.a.** If you would like to learn more about the EQUATOR Network go to: http://www.equator-network.org |
| **7.b.** How many times have you visited the EQUATOR Network website? |
|  |
| 1 - 10 |
| 11 + |
| **Don't know** |

### 5. Beliefs about reporting quality

| **8.** Do you think that information (such as details of an intervention or treatment) omitted from journal articles is a: |
| --- |
| **8.a.** common occurrence? -- Indicate |
| **No** |
| Yes |
| **8.b.** significant problem for "consumers" of research reports and articles (i.e. e.g. systematic reviewers, policy makers, clinicians)? -- Indicate |
| **No** |
| Yes |

### 6. Beliefs about reporting guidelines

| **9.** When do you think a reporting guideline should be used? |
| --- |
| **During all stages of a research project** |
| Only when required by a journal |
| When designing a study |
| When required by a funding body |
| When writing up a study |

| **10.** Who do you think should use reporting guidelines? |
| --- |
| **All authors** |
| Lead author |
| Junior researchers |
| Journal editors |
| Peer reviewers |
| Students |

### 7. Awareness and perceptions of reporting guidelines

| **11.** Which reporting guidelines are you familiar with? |
| --- |
| **CONSORT** |
| CONSORT extensions |
| COREQ |
| MOOSE |
| PRISMA |
| REMARK |
| SQUIRE |
| STARD |
| STRICTA |
| STROBE |
| TREND |

### 8. Awareness and perceptions of reporting guidelines

| **12.** Are you aware of the Transparent Reporting of Evaluations with Non-randomized Designs (TREND) reporting guideline? |
| --- |
| No |
| **Yes** |
| **12.a.** When did you first learn of the TREND guideline? |
| **2004** |
| 2005 |
| 2006 |
| 2007 |
| 2008 |
| 2009 |
| 2010 |
| 2011 |
| 2012 |
| 2013 |
| **12.b.** How did you learn of the TREND guidelines? |
|  |
| **12.c.** What do you think are the strengths of TREND? |
|  |
| **12.d.** What do you think are the limitations of TREND? |
|  |
| **12.e.** Answer the following two questions using a scale of 1 - 5. How 'credible' do you think the development process of TREND was? where 1 is not very credible, and 5 is very credible |
| **1** |
| 2 |
| 3 |
| 4 |
| 5 |
| Don't know |
| Not important to me |
| **12.f.** How transparent do you think the development of TREND was? Where 1 is “not very transparent”, and 5 is “very transparent” |
| **1** |
| 2 |
| 3 |
| 4 |
| 5 |
| Don't know |
| Not important to me |

### 9. Perceptions of reporting guidelines

| **13.** Using a scale of 1 - 5, where 1 is "not very important" (or "useful" depending on the question) and 5 is "very important" (or "useful"). |
| --- |
| **13.a.** How important for improving the completeness of reporting of research do you think it is that authors follow reporting guidelines? -- Indicate level of importance or usefulness |
| **1 - not very** |
| 2 |
| 3 |
| 4 |
| 5 - very |
| Don't know |
| **13.b.** How important do you think reporting guidelines are to authors (in general) when writing their research for publication? -- Indicate level of importance or usefulness |
| **1 - not very** |
| 2 |
| 3 |
| 4 |
| 5 - very |
| Don't know |
| **13.c.** How important do you think it is to have a mechanism for giving feedback regarding the usefulness of a reporting guideline? -- Indicate level of importance or usefulness |
| **1 - not very** |
| 2 |
| 3 |
| 4 |
| 5 - very |
| Don't know |
| **13.d.** How useful do you think guidelines are in assisting authors to write-up their research for publication? -- Indicate level of importance or usefulness |
| **1 - not very** |
| 2 |
| 3 |
| 4 |
| 5 - very |
| Don't know |
| **13.e.** How useful do you think reporting guidelines are to authors when designing their study? -- Indicate level of importance or usefulness |
| **1 - not very** |
| 2 |
| 3 |
| 4 |
| 5 - very |
| Don't know |

### 10. Perceptions of reporting guidelines

| **14.** For which sections of a research paper/article do you think reporting guidelines are: |
| --- |
| **14.a.**  most useful when reporting your research? -- (Mandatory response) |
| **Title** |
| Abstract |
| Introduction |
| Method |
| Results |
| Discussion |
| Don't know |
| **14.a.i.**  most useful when reporting your research? -- (Optional) |
| Title |
| Abstract |
| Introduction |
| Method |
| Results |
| Discussion |
| **14.a.ii.**  most useful when reporting your research? -- (Optional) |
| Title |
| Abstract |
| Introduction |
| Method |
| Results |
| Discussion |
| **14.b.**  least useful when reporting your research? -- (Mandatory response) |
| **Title** |
| Abstract |
| Introduction |
| Method |
| Results |
| Discussion |
| Don't know |
| **14.b.i.**  least useful when reporting your research? -- (Optional) |
| Title |
| Abstract |
| Introduction |
| Method |
| Results |
| Discussion |
| **14.b.ii.**  least useful when reporting your research? -- (Optional) |
| Title |
| Abstract |
| Introduction |
| Method |
| Results |
| Discussion |

### 11. Perceptions of reporting guidelines

| **15.** When do you think revisions should be made to reporting guidelines? |
| --- |
| **every 1-2 years** |
| every 3-4 years |
| every 5-6 years |
| every 7-10 years |
| review triggered by new ideas about reporting |
| review triggered by research findings |

### 12. Perceptions of reporting guidelines

| **16.** Do you think the use of reporting guidelines should be... |
| --- |
| **16.a.** a pre-condition of funding? -- Indicate |
| **No** |
| Yes |
| **16.b.** a pre-condition of publication? -- Indicate |
| **No** |
| Yes |

| **17.** Who do you think should monitor or enforce the use of reporting guidelines? |
| --- |
| employers |
| authors |
| ethics committees |
| funding agencies |
| journal editorial or administrative staff |
| line managers/supervisors |
| peer reviewers |
| **self-monitor** |
| no one |

### 13. Use of reporting guidelines

| **18.** What would make you more likely to use reporting guidelines in the future? |
| --- |
| Employer requirement |
| Endorsements/recommendations from colleagues |
| Endorsements of reporting guidelines by journal(s) |
| Evidence showing increase chance of publication if used a reporting guideline |
| Evidence that shows use of guidelines improves reporting quality |
| Funding requirements |
| **Journal requirements** |

### 14. Use of reporting guidelines

| **19.** Which reporting guidelines have you used? |
| --- |
| CONSORT |
| CONSORT extensions |
| COREQ |
| MOOSE |
| PRISMA |
| REMARK |
| SQUIRE |
| STARD |
| **STRICTA** |
| STROBE |
| TREND |

| **20.** Where you are aware of a reporting guideline relevant to your type of study, how frequently do you use reporting guidelines? |
| --- |
| **for 100% of articles/reports I write** |
| for 80 - 99% of articles/reports I write |
| for 60 - 79% of articles/reports I write |
| for less than 60% articles I write |
| Only when a journal requires it |
| On occasions when agreed with colleagues |

### 15. Use of reporting guidelines

| **21.** |
| --- |
| **21.a.** When using a reporting guideline do you usually use the whole guideline or particular parts of it? -- Indicate (mandatory) |
| The whole reporting guideline |
| **Title** |
| Abstract |
| Introduction |
| Method |
| Results |
| Discussion |
| **21.a.i.** When using a reporting guideline do you usually use the whole guideline or particular parts of it? -- (optional) |
| Title |
| Abstract |
| Introduction |
| Method |
| Results |
| Discussion |
| **21.a.ii.** When using a reporting guideline do you usually use the whole guideline or particular parts of it? -- (optional) |
| The whole reporting guideline |
| Title |
| Abstract |
| Introduction |
| Method |
| Results |
| Discussion |

| **22.** What was/were your reason(s) for using reporting guidelines? |
| --- |
|  |

### 16. Use of reporting guidelines

| **23.** On a scale of 1 to 5, where 1 is "no agreement" and 5 is "strong agreement", please indicate the degree to which you agree with the following statements... |
| --- |
| **23.a.** Journals' instructions to authors are often ambiguous and confusing with regard to use of reporting guidelines. -- 1 - "no agreement" to 5 - "strong agreement" |
| **1** |
| 2 |
| 3 |
| 4 |
| 5 |
| Don't know |
| **23.b.** I consider the evidence base of respective reporting guidelines before deciding to use them. -- 1 - "no agreement" to 5 - "strong agreement" |
| **1** |
| 2 |
| 3 |
| 4 |
| 5 |
| Don't know |
| **23.c.** Transparency of the development process of guidelines is important to me when considering use of a reporting guideline. -- 1 - "no agreement" to 5 - "strong agreement" |
| **1** |
| 2 |
| 3 |
| 4 |
| 5 |
| Don't know |
| **23.d.** Using a reporting guideline takes too long. -- 1 -"no agreement" to 5 - "strong agreement" |
| **1** |
| 2 |
| 3 |
| 4 |
| 5 |
| Don't know |
| **23.e.** A reporting guideline is too prescriptive when writing up a study. -- 1 - "no agreement" to 5 - "strong agreement" |
| **1** |
| 2 |
| 3 |
| 4 |
| 5 |
| Don't know |
| **23.f.** Meeting the requirements of the journal is more important than using a reporting guideline. -- 1 - "no agreement" to '5 - "strong agreement" |
| **1** |
| 2 |
| 3 |
| 4 |
| 5 |
| Don't know |
| **23.g.** Journals should align their requirements with reporting guidelines. -- 1 - "no agreement" to 5 - "strong agreement" |
| **1** |
| 2 |
| 3 |
| 4 |
| 5 |
| Don't know |
| **23.h.** There aren't relevant reporting guidelines for my field of research. -- 1 - "no agreement" to 5 - "strong agreement" |
| **1** |
| 2 |
| 3 |
| 4 |
| 5 |
| Don't know |
| **23.i.** I have difficulty locating reporting guidelines relevant to my research. -- 1 - "no agreement" to 5 - "strong agreement" |
| **1** |
| 2 |
| 3 |
| 4 |
| 5 |
| Don't know |
| **23.j.** Reporting guidelines need to be complemented by other strategies (e.g. standardised requirements from journals to use reporting guidelines) to improve reporting completeness. -- 1 - "no agreement" to 5 - "strong agreement" |
| **1** |
| 2 |
| 3 |
| 4 |
| 5 |
| Don't know |

### 17. Use of reporting guidelines

| **24.** On a scale of 1 to 5 where 1 is "not important" and 5 is "very important", rate how important it is, when considering the use of a reporting guideline, that there is: |
| --- |
| **24.a.** A website with explanatory information -- 1 - "not important" to 5 - "very important" |
| **1** |
| 2 |
| 3 |
| 4 |
| 5 |
| Don't know |
| **24.b.** An "explanation and elaboration" document about the reporting guideline -- 1 - "not important" to 5 - "very important" |
| **1** |
| 2 |
| 3 |
| 4 |
| 5 |
| Don't know |
| **24.c.** Courses, workshops, or lectures to support the use of the reporting guideline -- 1 - "not important" to 5 - "very important" |
| **1** |
| 2 |
| 3 |
| 4 |
| 5 |
| Don't know |
| **24.d.** Endorsement of the reporting guidelines from journals that you might submit articles to -- 1 - "not important" to 5 - "very important" |
| **1** |
| 2 |
| 3 |
| 4 |
| 5 |
| Don't know |
| **24.e.** Endorsement of the reporting guideline from professional societies -- 1 - "not important" to 5 - "very important" |
| **1** |
| 2 |
| 3 |
| 4 |
| 5 |
| Don't know |
| **24.f.** Use of reporting guidelines by your peers -- 1 - "not important" ' to 5 - "very important" |
| **1** |
| 2 |
| 3 |
| 4 |
| 5 |
| Don't know |

### 18. Interview and Feedback

| **25.** Would you be willing to take part in a semi-structured interview about your views on reporting quality and reporting guidelines? |
| --- |
| **Yes** |
| No |
| **25.a.**  As this survey is completely anonymous, it is important that you write your name and email address in the space provided. Please write your name here: |
|  |
| **25.b.** Please write your email address here: |
|  |

| **26.** If you would like to be kept informed of the results of the project, please leave your email address here: |
| --- |
|  |

### 19. Suggestions

| **27.** We would be interested to know what additional comments or thoughts you might have about the use of reporting guidelines, how adherence to them could be improved, or how reporting completeness could be increased. |
| --- |
|  |

### Editor Survey

Page 1 of 18

**Welcome**

Welcome to this survey about reporting quality and guidelines. The purpose of this survey is to collect information about your views about the quality and completeness of reporting of research studies, and whether or not reporting guidelines might be an effective strategy to improve reporting. 

NOTE: For the purposes of this survey we are referring to reporting guidelines (e.g. CONSORT) which are independent of/external to your journal's requirements.

The survey has received ethics approval (reference: 15/06/159) from the Research Ethics Committee of the University of Exeter Medical School, United Kingdom. The study protocol has been published in BMJ Open and is available here: <http://bmjopen.bmj.com/content/2/6/e002073.full.pdf+html>

Funding for project was provided by the National Institute for Health Research (NIHR) Peninsula Collaboration for Leadership in Applied Health Research & Care (PenCLAHRC). For more information about PenCLAHRC go to: <http://clahrc-peninsula.nihr.ac.uk/>

Page 2 of 18

**Completing the survey**

The survey is completed anonymously, can be saved part way through and takes around 10 - 15 minutes to complete. Questions are mandatory unless marked otherwise.

*Note: once you have clicked on the CONTINUE button your answers are submitted and you cannot return to, review, or amend that page.*

**Data protection**
All data collected in this survey will be held anonymously and securely.

Cookies and personal data stored by your Web browser are not used in this survey.

**If you need assistance with any aspect of completing the survey or encounter errors with the program, please email t.fuller@exeter.ac.uk**

**Consent**

Before commencing the survey we would like to remind you that:

- participation is voluntary;
- you are free to withdraw from the project at any time;
- your data will be retained in secure storage;
- there is no financial compensation or incentive for participating in this project; and,
- the results of the project will be published but your anonymity will be preserved.

| **1.** I have read the project information sheet and give my informed consent to participate in the study ''Evaluating the impact and use of the Transparent Reporting of Evaluations with Non-randomised Designs (TREND) reporting guidelines.'' |
| --- |
| **Yes** |

*If "No", thank you for considering taking part in the study.  You can exit the survey by closing your browser or tab.*

### 2. Professional background

| **2.** Indicate which best describes you |
| --- |
| **I work only as a journal editor** |
| I spend half my time working as an editor and half in another role (e.g. academic, clinician) |
| I work more than .5 full time equivalent as an editor and am employed in another role (e.g. academic, clinician) |
| I work less than .5 full time equivalent and am employed in another role (e.g. academic, clinician) |

| **3.** What is/are your current job title(s)? |
| --- |
| Assistant editor |
| **Editor** |
| Senior editor |
| Editor in chief |
| Assoc. Research Fellow |
| Research Fellow |
| Lecturer |
| Senior Lecturer |
| Senior Research Fellow |
| Assoc. Prof. |
| Professor |

| **4.** Please provide an estimate of years of editorial experience. |
| --- |
|  |

| **5.** Please provide an estimate of years of research experience. |
| --- |
|  |

### 3. Professional background

| **6.** In which country are you employed? |
| --- |
| UK |

| **7.** What is your '''''primary''''' field of expertise? |
| --- |
| Epidemiology |
| Evidence synthesis/systematic reviews |
| Health services research |
| Medicine |
| Nursing |
| Physiotherapy |
| **Psychology** |
| Public Health |
| Research Methods |
| Social Work |
| Statistics |

| **8.** Are you a member of any professional organisations, societies or colleges? (e.g. COPE, ICMJE, WAME) |
| --- |
| No |
| **Yes** |
| **8.a.** Which professional organisations or societies are you member of? |
|  |

### 4. Reporting guideline endorsement

| **9.** Does your journal mention reporting guidelines such as CONSORT and TREND in its instructions to authors in addition to its own requirements for content? |
| --- |
| Don't know |
| No |
| **Yes** |
| **9.a.** Which guidelines are mentioned in the instructions to authors? |
| **CONSORT** |
| CONSORT extensions |
| COREQ |
| MOOSE |
| **PRISMA** |
| REMARK |
| SQUIRE |
| STARD |
| STRICTA |
| **STROBE** |
| **TREND** |
| **9.b.** What was/were the reason(s) for mentioning reporting guidelines in the instructions to authors? |
|  |
| **9.c.** What might make your journal more likely to refer to reporting guidelines in its instructions to authors in future? |
| Publisher requirement |
| Competitor journal promoting the use of reporting guidelines |
| Endorsements/recommendations from editorial colleagues |
| Evidence showing improved status or metrices if promoting or endorsing the use of a reporting guideline |
| Evidence that shows the use of guidelines improves reporting quality of manuscripts submitted to journals |
| My journal already promotes the use of reporting guidelines |
| Don't know |

### 5. EQUATOR Network

| **10.** Are you aware of the EQUATOR Network? |
| --- |
| No |
| **Yes** |
| **10.a.** If you would like to learn more about the EQUATOR Network go to: http://www.equator-network.org |
| **10.b.** How many times have you visited the EQUATOR Network website? |
| 0 |
| 1-10 |
| **11+** |
| Don't know |

### 6. Beliefs about reporting quality

| **11.** Do you think that information (such as details of an intervention or treatment) omitted from journal articles is a: |
| --- |
| **11.a.** common occurrence? -- Indicate |
| No |
| **Yes** |
| Don't know |
| **11.b.** significant problem for "consumers" of research reports and articles (i.e. e.g. systematic reviewers, policy makers, clinicians)? -- Indicate |
| No |
| **Yes** |
| Don't know |

### 7. Beliefs about reporting guidelines

| **12.** Who do you think should use reporting guidelines? |
| --- |
| **All authors** |
| Lead author |
| Junior researchers |
| **Journal editors** |
| **Peer reviewers** |
| Students |
| Don't know |

| **13.** When do you think authors should use a reporting guideline (such as CONSORT etc)? |
| --- |
| **During all stages of a research project** |
| **When required by a journal** |
| When designing a study |
| **When required by a funding body** |
| When writing up a study |
| Don't know |

| **14.** When do you think editors should use reporting guidelines such as CONSORT? |
| --- |
| **When required by a publisher** |
| **When considering to send a paper out for peer review** |
| After a paper is recommended for publication by reviewers |
| I don't think I need to use reporting guidelines such as CONSORT or TREND as the journal has its own guidelines for what authors should include in papers |
| Don't know |

### 8. Awareness and perceptions of reporting guidelines

| **15.** Which reporting guidelines are you familiar with? |
| --- |
| **CONSORT** |
| CONSORT extensions |
| COREQ |
| MOOSE |
| **PRISMA** |
| REMARK |
| SQUIRE |
| STARD |
| STRICTA |
| **STROBE** |
| None of the above |

### 9. Awareness and perceptions of reporting guidelines

| **16.** Are you aware of the Transparent Reporting of Evaluations with Non-randomized Designs (TREND) reporting guideline? |
| --- |
| No |
| **Yes** |
| **16.a.** When did you first learn of the TREND guideline? |
| 2004 |
| 2005 |
| 2006 |
| 2007 |
| 2008 |
| 2009 |
| 2010 |
| 2011 |
| **2012** |
| 2013 |
| **16.b.** How did you learn of the TREND guidelines? |
|  |
| **16.c.** What do you think are the strengths of TREND? |
|  |
| **16.d.** What do you think are the limitations of TREND? |
|  |
| **16.e.** Answer the following two questions using a scale of 1 - 5. How '''''credible''''' do you think the development process of TREND was? where '''''1 is not very credible''''', and '''''5 is very credible''''' |
| 1 |
| 2 |
| 3 |
| 4 |
| 5 |
| Don't know |
| **Not important to me** |
| **16.f.** How '''''transparent''''' do you think the development of TREND was? Where '''''1 is not very transparent''''', and '''''5 is very transparent''''' |
| 1 |
| 2 |
| 3 |
| 4 |
| 5 |
| Don't know |
| Not important to me |

### 10. Perceptions of reporting guidelines

| **17.** Using a scale of 1 - 5, where '''1 is "not very important"''' (or "useful" depending on the question) and '''5 is "very important"''' (or "useful"). |
| --- |
| **17.a.** How ''important'' do you think it is that your journal promotes the use of reporting guidelines to improve reporting quality (e.g. through including advice to follow them in the instructions to authors)? -- Indicate level of importance or usefulness |
| 1 - not very |
| 2 |
| 3 |
| 4 |
| **5 - very** |
| Don't know |
| **17.b.** How ''important'' for improving the completeness of reporting of research do you think it is that authors follow reporting guidelines? -- Indicate level of importance or usefulness |
| 1 - not very |
| 2 |
| 3 |
| 4 |
| **5 - very** |
| Don't know |
| **17.c.** How ''important'' do you think reporting guidelines and their checklists are to peer reviewers when reviewing and considering articles for publication? -- Indicate level of importance or usefulness |
| 1 - not very |
| 2 |
| 3 |
| 4 |
| **5 - very** |
| Don't know |
| **17.d.** How ''important'' do you think it is to have a mechanism for giving feedback to developers regarding the usefulness of a reporting guideline? -- Indicate level of importance or usefulness |
| 1 - not very |
| 2 |
| 3 |
| 4 |
| **5 - very** |
| Don't know |
| **17.e.** How ''useful'' do you think reporting guidelines are in assisting authors to write-up their research for publication? -- Indicate level of importance or usefulness |
| 1 - not very |
| 2 |
| 3 |
| 4 |
| **5 - very** |
| Don't know |
| **17.f.** How ''useful'' do you think reporting guidelines are to authors when ''designing'' their study? -- Indicate level of importance or usefulness |
| 1 - not very |
| 2 |
| 3 |
| 4 |
| **5 - very** |
| Don't know |

### 11. Perceptions of reporting guidelines

| **18.** When do you think revisions should be made to reporting guidelines? |
| --- |
| every 1-2 years |
| every 3-4 years |
| every 5-6 years |
| every 7-10 years |
| review triggered by new ideas about reporting |
| **review triggered by research findings** |
| don't know |

### 12. Perceptions of reporting guidelines

| **19.** Do you think the use of reporting guidelines by authors should be... |
| --- |
| **19.a.** a pre-condition of funding? -- Indicate |
| No |
| **Yes** |
| Don't know |
| **19.b.** a pre-condition of publication? -- Indicate |
| No |
| **Yes** |
| Don't know |

| **20.** Who do you think should monitor or enforce the use of reporting guidelines? |
| --- |
| **authors should self-monitor** |
| **ethics committees** |
| **funding agencies** |
| **journal editorial or administrative staff** |
| line managers/supervisors of authors |
| peer reviewers |
| no one |
| don't know |

### 13. Use of reporting guidelines

| **21.** On a scale of 1 to 5, where '''1 is "no agreement"''' and '''5 is "strong agreement"''', please indicate the degree to which you agree with the following statements... |
| --- |
| **21.a.** The editorial board should consider the evidence base of respective reporting guidelines before including a reference to them in the instructions for authors. -- '''1 - "no agreement"''' to '''5 - "strong agreement"''' |
| 1 |
| 2 |
| 3 |
| 4 |
| **5** |
| Don't know |
| **21.b.** Transparency of the development process of guidelines is important when considering use of a reporting guideline. -- '''1 - "no agreement"''' to '''5 - "strong agreement"''' |
| 1 |
| 2 |
| 3 |
| 4 |
| **5** |
| Don't know |
| **21.c.** Enforcing the use of a reporting guideline is/would be too time consuming. -- '''1 - "no agreement"''' to '''5 - "strong agreement"''' |
| 1 |
| 2 |
| 3 |
| 4 |
| **5** |
| Don't know |
| **21.d.** A reporting guideline is too prescriptive for authors when writing up a study. -- '''1 - "no agreement"''' to '''5 - "strong agreement"''' |
| 1 |
| 2 |
| 3 |
| 4 |
| **5** |
| Don't know |
| **21.e.** Meeting the requirements of the journal is more important than using a reporting guideline. -- '''1 - "no agreement"''' to '''5 - "strong agreement"''' |
| 1 |
| 2 |
| 3 |
| 4 |
| **5** |
| Don't know |
| **21.f.** There aren't relevant reporting guidelines for my journal's field of research. -- '''1 - "no agreement"''' to '''5 - "strong agreement"''' |
| 1 |
| 2 |
| 3 |
| 4 |
| **5** |
| Don't know |

### 14. Use of reporting guidelines

| **22.** On a scale of 1 to 5 where '''1 is "not important"''' and '''5 is "very important"''', rate how important it is, when considering the use of a reporting guideline, that there is: |
| --- |
| **22.a.** A website with explanatory information about the reporting guideline -- '''1 - "not important"''' to '''5 - "very important"''' |
| 1 |
| 2 |
| 3 |
| 4 |
| **5** |
| Don't know |
| **22.b.** An "explanation and elaboration" document about the reporting guideline -- '''1 - "not important"''' to '''5 - "very important"''' |
| 1 |
| 2 |
| 3 |
| 4 |
| **5** |
| Don't know |
| **22.c.** Courses, workshops, or lectures to support the use of the reporting guideline -- '''1 - "not important"''' to '''5 - "very important"''' |
| 1 |
| 2 |
| 3 |
| 4 |
| **5** |
| Don't know |
| **22.d.** Endorsement of the reporting guideline from professional societies -- '''1 - "not important"''' to '''5 - "very important"''' |
| 1 |
| 2 |
| 3 |
| 4 |
| **5** |
| Don't know |
| **22.e.** Use of reporting guidelines by your peers/other editors of journals in your field -- '''1 - "not important"''' to '''5 - "very important"''' |
| 1 |
| 2 |
| 3 |
| 4 |
| **5** |
| Don't know |

### 15. Interview and Feedback

| **23.** Would you be willing to take part in a semi-structured interview about your views on reporting quality and reporting guidelines? |
| --- |
| **Yes** |
| No |
| **23.a.** ''As this survey is completely anonymous, it is important that you write your name and email address in the space provided so that we can contact you about arranging an interview.'' Please write your name here: |
|  |
| **23.b.** Please write your email address here: |
|  |

| **24.** If you would like to be kept informed of the results of the project, please leave your email address here: |
| --- |
|  |

### 16. Suggestions

| **25.** We would be interested to know what additional comments or thoughts you might have about the use of reporting guidelines, how adherence to them could be improved, or how reporting completeness could be increased. |
| --- |
|  |
