# Supplementary material for: What Affects Authors’ and Editors’ Use of Reporting Guidelines? Findings from an Online Survey and Qualitative Interviews
Source: PLoS One. 2015 Apr 15;10(4):e0121585. doi: 10.1371/journal.pone.0121585 (PMC4398362; doi:10.1371/journal.pone.0121585)
Supplement: S4 File — (DOCX) [file pone.0121585.s004.docx]

**S4 File**

**Table A in S4 File.** Consolidated criteria for reporting qualitative research (COREQ): a 32-item checklist for interviews and focus groups

| **No** | **Item** | **Guide questions/description** |
| --- | --- | --- |
| Domain 1: Research team and reflexivity |  |  |
| Personal Characteristics |  |  |
| 1. | Interviewer/facilitator | Which author/s conducted the interview or focus group? REPORTED PG 9. (TF CONDUCTED INTERVIEWS) |
| 2. | Credentials | What were the researcher's credentials? E.g. PhD, MD. REPORTED IN SUPPLEMENTARY METHODS FILE. |
| 3. | Occupation | What was their occupation at the time of the study? REPORTED IN SUPPLEMENTARY METHODS FILE. |
| 4. | Gender | Was the researcher male or female? REPORTED IN SUPPLMENTARY METHODS FILE |
| 5. | Experience and training | What experience or training did the researcher have? REPORTED IN SUPPLEMENTARY METHODS FILE |
| Relationship with participants |  |  |
| 6. | Relationship established | Was a relationship established prior to study commencement? REPORTED IN SUPPLEMENATRY METHODS FILE |
| 7. | Participant knowledge of the interviewer | What did the participants know about the researcher? e.g. personal goals, reasons for doing the research REPORTED IN SUPPLEMENATRY METHODS FILE |
| 8. | Interviewer characteristics | What characteristics were reported about the interviewer/facilitator? e.g. Bias, assumptions, reasons and interests in the research topic REPORTED IN SUPPLEMENATRY METHODS FILE |
| Domain 2: study design |  |  |
| Theoretical framework |  |  |
| 9. | Methodological orientation and Theory | What methodological orientation was stated to underpin the study? e.g. grounded theory, discourse analysis, ethnography, phenomenology, content analysis Reported page 10. |
| Participant selection |  |  |
| 10. | Sampling | How were participants selected? Reported page 9. |
| 11. | Method of approach | How were participants approached? REPORTED IN SUPPLEMENATRY METHODS FILE |
| 12. | Sample size | How many participants were in the study? REPORTED ON PAGE 9, 11. |
| 13. | Non-participation | How many people refused to participate or dropped out? Reasons? REPORTED IN SUPPLEMENTARY RESULTS FILE |
| Setting |  |  |
| 14. | Setting of data collection | Where was the data collected? e.g. home, clinic, workplace REPORTED IN SUPPLEMENATRY METHODS FILE |
| 15. | Presence of non-participants | Was anyone else present besides the participants and researchers? REPORTED IN SUPPLEMENATRY METHODS FILE |
| 16. | Description of sample | What are the important characteristics of the sample? REPORTED ON PAGE 11 |
| Data collection |  |  |
| 17. | Interview guide | Were questions, prompts, guides provided by the authors? Was it pilot tested? REPORTED PAGE 10 |
| 18. | Repeat interviews | Were repeat interviews carried out? If yes, how many? REPORTED IN SUPPLEMENATRY METHODS FILE |
| 19. | Audio/visual recording | Did the research use audio or visual recording to collect the data? REPORTED PAGE 9 |
| 20. | Field notes | Were field notes made during and/or after the interview or focus group? REPORTED IN SUPPLEMENATRY METHODS FILE |
| 21. | Duration | What was the duration of the interviews or focus group? REPORTED IN SUPPLEMENATRY METHODS FILE |
| 22. | Data saturation | Was data saturation discussed? REPORTED IN SUPPLEMENATRY METHODS FILE |
| 23. | Transcripts returned | Were transcripts returned to participants for comment and/or correction? REPORTED PAGE 9 |
| Domain 3: analysis and findings |  |  |
| Data analysis |  |  |
| 24. | Number of data coders | How many data coders coded the data? REPORTED PAGE 10. |
| 25. | Description of the coding tree | Did authors provide a description of the coding tree? REPORTED PAGE 10 |
| 26. | Derivation of themes | Were themes identified in advance or derived from the data? REPORTED PAGE 10 |
| 27. | Software | What software, if applicable, was used to manage the data? REPORTED PAGE 10 |
| 28. | Participant checking | Did participants provide feedback on the findings? REPORTED IN SUPPLEMENATRY METHODS FILE |
| Reporting |  |  |
| 29. | Quotations presented | Were participant quotations presented to illustrate the themes / findings? Was each quotation identified? REPORTED FROM PAGE 17 THROUGHOUT THE RESULTS SECTION. |
| 30. | Data and findings consistent | Was there consistency between the data presented and the findings? REPORTED IN RESSULTS SECTION PAGE17 ONWARDS |
| 31. | Clarity of major themes | Were major themes clearly presented in the findings? REPORTED PAGE 15 |
| 32. | Clarity of minor themes | Is there a description of diverse cases or discussion of minor themes? REPORTED THROUGHOUT RESULTS – PAGE 15. |
