# Supplementary material for: What Affects Authors’ and Editors’ Use of Reporting Guidelines? Findings from an Online Survey and Qualitative Interviews
Source: PLoS One. 2015 Apr 15;10(4):e0121585. doi: 10.1371/journal.pone.0121585 (PMC4398362; doi:10.1371/journal.pone.0121585)
Supplement: S5 File — (DOCX) [file pone.0121585.s005.docx]

**S5 File**

# Online survey participant recruitment and response rate

**Table A in S5 File** Response rate for authors invited to complete the survey on their use and perceptions of TREND and other reporting guidelines

|  |  | Authors who cited use of TREND | | | Authors of papers who could have used TREND but did not report doing so* | | |  |
| --- | --- | --- | --- | --- | --- | --- | --- | --- |
|  |  | Corresponding author | | Second contactable author | Corresponding author | | Second contactable author | Total |
| Invited |  | 44 | | 39 | 47 | | 43 | 173 |
| Missing | Unable to locate current contact details | 0 | | 5 | 0 | | 3 | 8 |
|  | Authors on multiple papers and already invited | 3 | | 2 | 0 | | 1 | 6 |
|  | Single authored paper | 0 | | 1 | 0 | | 0 | 1 |
| Response |  | 29 total** | | | 27 total** | | | 56 |
| Declined |  | 0 | 0 | | 0 | 1 | | 1 |
| Response rate |  | 35%  (29/83) |  | | 30%  (27/90) |  | | 32%  (56/173) |

*Participants were recruited from an earlier study evaluating the impact of TREND [[1](#_ENREF_1)] and included authors who had and had not reported using TREND respectively to guide the write-up of their study.

**Totals are shown as it is not possible to determine, within groups, who responded.

One author actively declined to participate, indicating that they did not think that they would have any valuable information to contribute to the study.

**Table B in S5 File** Response rate of Journal editorial staff invited to participate in Study Two.

|  |  | **Editors in chief** | **Second contactable editor** | **Combined** |
| --- | --- | --- | --- | --- |
| **Invited** |  | **83** | **76** | **159** |
| **Missing** | No email found | 0 | 2 | 2 |
|  | No associate or assistant editorial staff listed. | 0 | 5 | 5 |
| **Declined** |  | 2 | 9 | 11 |
| **Responded** |  |  |  | **43** |
| **Response rate** |  |  |  | **27.0% (43/159)** |

Note: there were 9 incomplete surveys not included in the analysis

**Table C in S5 File** Editors’ beliefs about who should use reporting guidelines

|  | N | Percentage of sample |
| --- | --- | --- |
| All authors | 34 | 79 |
| Peer reviewers | 30 | 70 |
| Journal editors | 28 | 65 |
| Students | 16 | 37 |
| Junior researchers | 15 | 35 |
| Lead author | 14 | 33 |
| Don't know | 5 | 12 |
| Other | 3 | 7 |

**Table D in S5 File** Editors’ beliefs about when authors should use reporting guidelines

|  | Responses | Percentage of sample |
| --- | --- | --- |
| All stages | 27 | 63 |
| When designing the study | 20 | 47 |
| When writing up the study | 20 | 47 |
| When required by journal | 12 | 28 |
| When required by funding body | 10 | 23 |
| Don't know | 5 | 12 |
| Other | 5 | 12 |

**Table E in S5 File** Editors’ beliefs about when editors’ should use reporting guidelines

|  | Responses | Percentage of sample |
| --- | --- | --- |
| When sending out for peer review | 25 | 58.1% |
| When required by publisher | 8 | 18.6% |
| Other | 8 | 18.6% |
| After a paper is recommended for publication | 8 | 18.6% |
| Don't know | 6 | 14.0% |
| Journal has its own guidelines | 2 | 4.7% |

Fifty-eight per cent of editors indicated that they thought the use of reporting guidelines should not be a pre-requisite for funding but 54% did view it as a pre-condition for publication.

**Table F in S5 File** Authors’ level of agreement with statements relating to use of reporting guidelines on scale of 1 (no agreement) – 5 (strong agreement), with 3 being “neutral”.

| **Question** | **N** | **Median** | **Min** | **Max** |
| --- | --- | --- | --- | --- |
| Journals' instructions to authors are often ambiguous and confusing with regard to use of reporting guidelines. | 29 | 3 | 1 | 5 |
| I consider the evidence base of respective reporting guidelines before deciding to use them. | 34 | 2 | 1 | 5 |
| Transparency of the development process of guidelines is important to me when considering use of a reporting guideline. | 32 | 3 | 1 | 5 |
| Using a reporting guideline takes too long. | 33 | 2 | 1 | 5 |
| A reporting guideline is too prescriptive when writing up a study | 33 | 2 | 1 | 5 |
| Meeting the requirements of the journal is more important than using a reporting guideline. | 33 | 3 | 1 | 5 |
| Journals should align their requirements with reporting guidelines. | 33 | 4.5 | 3 | 5 |
| There aren't relevant reporting guidelines for my field of research. | 31 | 1 | 1 | 3 |
| I have difficulty locating reporting guidelines relevant to my field of research | 34 | 2 | 1 | 5 |
| Reporting guidelines need to be complemented by other strategies (e.g. standardised requirements from journals to use reporting guidelines) to improve reporting completeness. | 33 | 4 | 1 | 5 |

Note: numbers vary due to the number of people indicating “don’t know” responses.

**Table G in S5 File** Authors’ ratings of importance of statements potentially influencing use of reporting guidelines (on scale of 1 – 5, higher scores indicate greater importance of item).

| **Statement** | **N** | **Median** | **SD** |
| --- | --- | --- | --- |
| Importance of website with explanatory info for reporting guideline | 34 | 4 | 1.1 |
| An "explanation and elaboration" document about the reporting guideline | 34 | 4 | 1.2 |
| Courses, workshops, or lectures to support the use of the reporting guideline | 34 | 3 | 1.2 |
| Endorsement of the reporting guidelines from journals that you might submit articles to | 34 | 4 | 1.0 |
| Importance of endorsement from professional societies | 33 | 4 | 1.0 |
| Use of reporting guidelines by your peers | 34 | 4 | 1.1 |

Reference

1. Fuller T, Pearson M, Peters J, Anderson R (2014) Impact of the Transparent Reporting of Evaluations With Nonrandomized Designs Reporting Guideline: Ten Years On. American Journal of Public Health: e1-e8.
